# Neural correlates of foreign speech imitation: The effects of age and music

**DOI:** 10.1162/IMAG.a.75

**Published:** 2025-07-17

**Authors:** Xiaohui Yan, Jiaqi Mao, Zixin Ma, Kyle Perkins, Weizheng Li, Yang Wang, Fan Cao

**Affiliations:** Department of Psychology, the University of Hong Kong, Hong Kong, China; BCBL Basque Center on Cognition, Brain and Language, Donostia, Gipuzkoa, Spain; retired professor, Florida International University, Miami, FL, United States; Department of Psychology, Sun Yat-Sen University, Guangzhou, China

**Keywords:** representational similarity, foreign speech imitation, musicians, speech production

## Abstract

Adult learners of a foreign speech are often marked by having a foreign accent; however, children and adults with singing training tend to have better pronunciations than adults without music training. The assimilation hypothesis proposes that people tend to assimilate foreign speech to native speech during perception and production, which may explain foreign accent. Unfortunately, the neural mechanisms underlying the age and music effects are still unclear. In this study, we compared brain activation patterns in three groups of participants, namely, children, adults with singing training, and adults without music training (control adults) during native (Chinese) and foreign speech (Spanish) imitation with each word repeated three times. We found greater representational similarity between Chinese and Spanish in both groups of adults than in children during both speech perception and production, supporting the assimilation hypothesis. Furthermore, we found group-specific effects for the similarity between different times of imitation, suggesting different mechanisms. Specifically, control adults showed greater similarity between different times of Spanish word imitation than the other two groups in the medial orbital frontal cortex involved in adaptive learning/memory; children showed greater similarity than the other two groups in the bilateral inferior premotor/postcentral gyri involved in sensorimotor learning; adults with singing training showed greater similarity than the other two groups in the left superior temporal gyrus involved in auditory feedback. It suggests that singing training facilitates reliance on auditory discrimination, while children rely on somatosensory and speech motor control to learn foreign speech sounds, implicating different mechanisms of age and singing training effects. Our results provide insights in understanding the neural mechanisms of age and music effects in foreign speech learning.

## 1 Introduction

Speech production requires complex motor control, involving more than 100 muscles to work in concert. Speech motor control develops early in life (Perrin & Venance, 2019; Wächter et al., 2009), making the acquisition of a second language speech easier and more native for children than adults, where foreign accents are common (Flynn & Manuel, 1991; Geschwind & Carterette, 1966; Hack et al., 2012; Long, 1990; Wolfe, 1967). According to Kuhl et al. (2005), the end of the critical period around 7 years old is characterized by a reduced cortical plasticity in the motor and auditory circuits, along with lower proficiencies in foreign speech phonetic discrimination and speech production. Although reduced neural plasticity marks the end of the critical period, the specific underlying neural mechanisms of how learning foreign speech differs between children and adults remain unclear.

The Directions into Velocities of Articulators (DIVA) model provides a framework for understanding the neural mechanisms underlying foreign speech imitation (Tourville & Guenther, 2011), which is essentially a process of adjusting motor control according to somatosensory and auditory feedback. According to the DIVA model (Tourville & Guenther, 2011), a feedforward control system is responsible for projecting motor commands for executing articulatory gestures. At the same time, a feedback control system generates somatosensory expectations of the articulatory gestures, and compares incoming somatosensory and auditory feedbacks with sensory expectations. If error signals are detected, corrective motor commands are sent to the motor cortex (Guenther & Vladusich, 2012; Tourville & Guenther, 2011). Specifically, in the motor cortex, the middle precentral gyrus is a larynx area which directly controls muscles in the vocal-fold, operation of which determines the opening and closing of the glottal space, as well as tensing and relaxing of vocal folds, modulating the pitch. The inferior precentral gyrus is involved in lip and tongue movement control. Lesion studies found that the middle and inferior precentral gyrus are the most consistent regions associated with foreign accent syndrome in stroke patients (Higashiyama et al., 2021). While the middle and inferior precentral gyrus are involved in producing acquired speech sounds, the striatum, thalamus, and premotor cortex are more involved in vocal learning of novel speech sounds, according to Jarvis (2004, 2006) based on research of songbirds and humans. Simmonds (2015) further suggested that the vocal learning pathway (e.g., the striatum) becomes inactive too early during vocal learning, and the motor cortex for producing acquired speech sounds is involved instead, which may be why there is foreign accent in late second language learners.

Unfortunately, no studies have compared the real-time brain mechanisms underlying foreign speech imitation in children and adults. Previous studies have compared native and nonnative vowels imitation in adults (Carey, Miquel et al., 2017; Klein et al., 2006), as well as adults with higher L2 aptitude and those with lower L2 aptitude during L1 and L2 production (Hu, Ackermann et al., 2013). A few studies also concerned how age of acquisition affects speech production in second language (Berken et al., 2015; Berken, Gracco, et al., 2016; Frenck-Mestre et al., 2005). Two of them found that early bilinguals tend to show greater activation and greater grey matter volume in the putamen than late bilinguals (Berken, Gracco, et al., 2016; Frenck-Mestre et al., 2005), while another study found greater activation in the inferior frontal gyrus in early bilinguals than late bilinguals during speech production of L2 (Berken, Chai, et al., 2016). However, these studies exclusively scanned adults, with half being early bilinguals and half being late bilinguals, examining L2 as a long-term learning effect, rather than the real-time neural underpinnings of foreign speech acquisition.

A few studies have examined the real-time brain activations of adults learning new languages. One study found greater activation in the anterior insula and inferior frontal gyrus when learning to speak a new language compared to native speech production, especially in the first 10 min of speaking the new language (Moser et al., 2009). Training studies examined brain activation during non-native speech sound perception (Golestani & Zatorre, 2004) and production (Simmonds et al., 2014) before and after training. It was found that more efficient processing in the frontal speech areas was correlated with greater success in foreign phonetic identification (Golestani & Zatorre, 2004). Simmonds et al. (2014) found reduced activation in the anterior striatum over time both within and between scanning sessions, suggesting that the striatum becomes inactive too early during vocal learning. However, no studies have directly compared the learning process of foreign speech imitation between children and adults to understand why children have a reduced foreign accent.

One hypothesis for the existence of foreign accents in adults when learning a second language is that they use their first language as reference. Some previous studies have shown assimilation of foreign speech sounds to native sounds during perception in adult learners (Best & Tyler, 2007; Best et al., 2001).This inaccurate speech representation further causes failure in speech motor control during production (Ingram & Park, 1997). On the other hand, children confront less interference from native language. For example, Baker et al. (2008) found that children aged 7 to 14 years old performed better at distinguishing similar native and foreign speech sounds than adults. One possible reason for the less assimilation to the native speech in children than in adults is that the native speech representation system is still under development in children (Zevin, 2012). In fact, the representation of native speech sound categories shows a significant development for the age range of 6–12 years and 12–18 years (McMurray et al., 2018).

However, no neuroimaging evidence is available yet to support this assimilation hypothesis. Moreover, it is unknown whether the greater similarity between foreign speech sounds and native speech sounds in adults than in children exists only in perception or extends to production as well. In the current study, we compared the brain activation patterns of native Chinese-speaking children (aged 9–10) and adults in Spanish speech imitation. The task mimicked a naturalistic speech learning situation in which each Spanish word was repeated three times, with the participant repeating it after each presentation. Under this paradigm, we aimed at comparing how children and adults are different during this foreign speech learning process. We expect greater similarity between Chinese and Spanish representation in adults than in children and faster decline of the striatum in adults than in children during Spanish speech learning.

Furthermore, about 5–15% of the population have the ability to achieve native-like speech, even when the age of acquisition is late (Abrahamsson & Hyltenstam, 2008; Birdsong, 2005; Flynn & Manuel, 1991; Wells, 1985). It has been redundantly documented that there is cross-domain transfer from musical expertise to speech perception and production (Jekiel & Malarski, 2021; Milovanov & Tervaniemi, 2011; Weiss et al., 2015; Wong et al., 2007; Zuk et al., 2013), which could be explained by the common neural correlates involved in musical and speech processing in the auditory pathway and motor cortex (Ozdemir et al., 2006). Moreover, the difference in musical nodes is more trivial than that in speech phonemes, leading to the fact that musical abilities can be transferred to speech abilities. One study showed that musical ability predicted L2 phonological ability (both receptive and productive) even after controlling for other factors, but did not account for the unique variance in L2 syntax or lexical knowledge (Slevc & Miyake, 2006). Another study found that musical experience is correlated with greater pronunciation accuracy of English vowels after speech therapy for accent reduction (Jekiel & Malarski, 2021).

Unlike musical instrument training, which mainly fosters speech perception, vocal training is beneficial for both speech perception and speech production, as singing not only enhances individuals’ pitch discrimination, but also trains the vocal motor apparatus necessary for pitch production. As illustrated in a study by Christiner and Reiterer (2015), in which three groups of German adults (i.e., singers, instrumentalists, non-musicians) were asked to imitate sentences of a foreign language (i.e., Hindi), both singers and instrumentalists had higher performances than non-musicians in mimicking the Hindi utterances. In addition, as vocalists had more precise vocal control than instrumentalists, singers outperformed instrumentalists in the Hindi sentence imitation task. Therefore, singing may be a strong predictor of good pronunciation skills in foreign speech imitation.

Neurologically, researchers have found that professional singers tend to have a greater volume in the ventral primary somatosensory cortex, rostral supramarginal gyrus, and auditory cortex than non-musicians (Kleber et al., 2017). In addition, larger volumes of arcuate fasciculus, which is a fiber tract connecting temporal areas and prefrontal regions, were found in musicians than non-musicians (Halwani et al., 2011). These brain structural changes in musicians were argued to play a significant role in speech production (Halwani et al., 2011). However, no published studies have compared brain activation patterns during actual foreign speech learning in singers and non-musicians to understand the brain differences for singers to outperform non-musicians.

In the current study, we recruited adults with professional vocal music training for more than 2 years and we planned to compare the foreign speech learning mechanisms in the brain in singer adults, children and adults without music training (control adults) to understand why adults with vocal training and children have advantages than control adults in foreign speech learning. We expect singers to have more accurate representations than control adults in the feedforward motor control areas, including the key vocal learning regions in the striatum, or somatosensory and auditory feedback areas.

## 2 Method

### 2.1 Participants

We recruited 32 adults without music background (i.e., control adults) (mean age 22.6, range 19–31), 20 adults with vocal singing training for at least 2 years (mean age 19, range 18–24), and 20 children without music training (mean age 10.3, range 10–11) in the local city. The adults with singing training were recruited from a local music college. The demographic information of the participants is presented in Table 1. The adults with singing training had 4.08 years of professional vocal training on average (range: 2–9 years, std: 2 years), with age of vocal training onset being 7.95 years old on average (range: 6–17 years old, std: 3.75 years old). The two adult groups were matched on education and English pronunciation according to a native English speaker’s rating on each participant’s language sample (t(50) = 0.446, *p* = .657). All participants also met the following criteria: (a) native Chinese speakers with English as their L2; (b) never learned Spanish, French, Portuguese, or Italian for more than 3 months; (c) free of medical implants and other metal accessories; (d) free of claustrophobia, hearing disorders, attention deficit hyperactivity disorder (ADHD), other developmental disabilities, neurological disease, and psychiatric disorders; and (e) right-handed. The present study was approved by the ethics committee at the local university. Informed consent was obtained from all participants/parents of participants before data collection.

**Table 1.** Demographic information and behavioral tests results in each group of participants.

	Control adults	Adults with singing training	Children
*N*	32 (10 M)	20 (3 M)	20 (6 M)
Age (years)	22.6 (3.3) [19-31]	19.9 (1.5) [18-24]	10.3 (0.6) [10-11]
Rhyming judgment	36.8 (2.5)	35.3 (3.8)	31.0 (4.9)
Initial sound Deletion	26.5 (4.3)	23.9 (7.3)	15.5 (8.5)
Digit span (forward)	9.0 (1.4)	9.6 (1.3)	8.1 (1.3)
Digit span (backward)	6.4 (2.2)	6.2 (2.4)	4.7 (1.3)
AMMA (percentile)	61.4 (27.4)	80.5 (15.8)	-

Numbers in the parenthesis are standard deviations, and in the brackets are ranges.

M: males; AMMA: Advanced Measures of Music Audition.

### 2.2 Sensitivity analysis

Sensitivity analysis was conducted using Gpower 3.19. In order to achieve α = 0.001 and a statistical power of 95%, our current sample size would need an effect size of 1.51 for the comparison between control adults and children, 1.71 for the comparison between children and adults with singing training, and 1.49 for the comparison between control adults and adults with singing training. In the whole-brain analysis, our actual effect size is 3.09 (https://www.sdmproject.com/utilities/?show=Statistics) for all group comparisons when the voxel-level threshold was set at *p* < .001, which is much larger than needed.

### 2.3 Procedures

#### 2.3.1 Behavioral tasks

Several behavioral tests were administered before the fMRI scanning. A pseudoword rhyming judgment test and an initial sound deletion test were included to test phonological awareness. The pseudoword rhyming judgment test consists of 40 pairs of single-syllable English pseudowords, and participants were asked to determine if the two pseudowords in a pair rhymed or not. In the initial sound deletion test, participants were asked to listen to real English words and repeat it out loud without the initial sound. There were 30 words, including 10 single-syllable words, 10 two-syllable words, and 10 three-syllable words. Furthermore, we measured working memory using a digit span test in forward and reversed order. The digit span test was in Chinese, which is the first language of participants. In this test, experimenters explicitly read random digit strings with an increasing span. All participants were given the same tests.

In addition, we measured music aptitude in adult participants using the Advanced Measures of Music Audition (AMMA; Gordon, 1989). In this test, participants listened to 30 pairs of music audios, and after each pair of audio, they were asked to choose one from the following options: the two audios differ in tones, differ in rhythms, same, or not sure. There is a tonal score, a rhythm score, and a composite score in AMMA.

#### 2.3.2 fMRI task

In the speech imitation task, there was a Spanish run and a Chinese run that were counterbalanced across participants. For the Spanish run, there were 28 Spanish real words (15 two-syllable words, 10 three-syllable words, and 3 four-syllable words), and for the Chinese run, there were 28 Chinese pseudo-words with syllable numbers matched with the Spanish words. Chinese pseudo-words were used in order to avoid semantic activation in the native language but not in the foreign language. In both runs, participants were asked to listen to and repeat each word/pseudo-word three times consecutively. Audio stimuli played in the scanner were recorded by a native female speaker in Spanish and Chinese respectively. A 5-min practice session was conducted prior to the scanning.

As illustrated in Figure S1, a red cross and an audio word/pseudoword were presented for 1500 ms. Then, the participant was asked to imitate the word/pseudoword they just heard in the next 1500 ms (t1). After a “jitter” screen which was presented for 1000–5000 ms (3000 ms at average), the same word was played again for 1500 ms and the participant was asked to imitate the word for the second time (t2). The same procedure was repeated again for the third time (t3). After the third “jitter” screen, a red cross was displayed for 3000 ms without auditory stimulus, which served as the baseline, followed by the fourth “jitter”. Then, the next trial started. After the fMRI scanning, participants were asked to imitate the Spanish speech again outside the scanner in order to have a high-quality recording of their pronunciation.

### 2.4 fMRI data acquisition

The fMRI images were acquired using a 3T Siemens Prisma MRI scanner. Participants lay down in the scanner with a standard 20-channel head coil, and two foam pads were used to help reduce head movement. Before they entered the scanner, a mock scanner was used for practicing speaking with limited head movement. During scanning, a real-time monitoring of head movement was conducted, and participants were reminded to keep their head still while talking during the break between runs if the head movement was large. A single-shot echo planar imaging (EPI) sequence was adopted to collect functional BOLD signals, with an interleaved acquisition from bottom to top for each volume (repetition time (TR) = 2000 ms, echo time (TE) = 20.0 ms, flip angle = 80°, matrix size = 128 × 128, field of view (FOV) = 220 mm, slice thickness = 3.0 mm, number of slices = 34, voxel size =1.7 × 1.7 × 3.0 mm^3^). There were 348 volumes collected for each run. High-resolution structural T1-weighted 3D images (MPRAGE) were also acquired (TR = 2300 ms, TE = 3.24 ms, TI = 900 ms, flip angle = 9°, matrix size = 256 × 256, FOV = 260 mm, slice thickness = 1.0 mm, number of slices = 160).

### 2.5 Data analysis

#### 2.5.1 Acoustic analysis

##### 2.5.1.1 Voice onset time (VOT) analysis

As a behavioral indicator of the in-scanner task performance, we measured the voice onset time (VOT) of /b/ and /d/ in seven Spanish words using Praat (Boersma, 2001). The VOT is the time interval between a plosive consonant release and voicing onset. The seven Spanish words (“bebé”, “bueno”, “brazo”, “dado”, and “difícil”) we chose contained voiced stops (/b/ and /d/ in Spanish) in which their onsets of phonation occur before the consonant release, resulting in a negative VOT. Figure S2 illustrates the measurement of VOT in Praat.

##### 2.5.1.2 Formant frequency analysis

The formant frequency analysis included the same seven Spanish words as the VOT analysis, covering all vowels in Spanish (/a/, /e/, /i/, /o/, and /u/). In order to quantify the performance on Spanish vowels’ imitation, the first formant (F1) and second formant (F2) of each vowel were calculated in Praat. The F1 and F2 are the first two resonating frequencies in a vowel’s pronunciation, with F1 indicating the opening of lips and F2 indicating the tongue’s position (Wood, 1982). The individual formant values were normalized using the R package NORM (Thomas & Kendall, 2007), in order to eliminate the influence of gender and age (Fabricius et al., 2009).

Two independent researchers who were blinded about the participants’ information calculated the VOT and frequency formants, and their evaluation results were correlated (*r* = .856, *p* < .001 for the VOT; *r* = .982, *p* < .001 for the frequency formant). We averaged their results to serve as the final score. The VOT and formant values of each consonant and vowel were averaged across all words and entered into a repeated-measure ANOVA of group (control adults, adults with singing training, children) by time (1^st^, 2^nd^, 3^rd^) for further analysis.

#### 2.5.2 fMRI images preprocessing

fMRI data preprocessing was conducted using DPARSF 4.3 (Yan et al., 2016; http://rfmri.org/DPARSF). First of all, slice timing was performed to correct timing difference of the interleaved slices with the middle slice as the reference. Next, functional images were aligned to the first volume to correct head movement. We used ART (Artifact Detection Tools, https://www.nitrc.org/projects/artifact_detect) to detect head movements that exceeded 3 mm for translations or 3° for rotations for each participant. We found that 4 participants (2 from the adults without music training group and 2 from the children group) had excessive head movements for less than 8 volumes across all sessions. Considering that the affected data points were less than 2% of the total data in each participant, we kept these participants and repaired the affected volumes in ArtRepair (https://www.nitrc.org/projects/art_repair/), using the interpolated values from neighboring time points to replace the affected time points. We also deleted two participants due to extensive head movement (1 from the adults without music training group and 1 from the adults with singing training group). The current sample size was after eliminating these two participants. Then, T1-weighted structural images were co-registered to the realigned functional images for each individual. A filter was applied so that only signals above 0.01 Hz were kept. The images were segmented into gray matter, white matter, and cerebrospinal fluid, before being normalized to the Montreal Neurological Institute (MNI) space. Finally, the normalized images were detrended by regressing out the nuisances using a Friston 24-parameter model (Friston et al., 1996).

#### 2.5.3 Representational similarity analysis (RSA)

RSA was conducted to calculate the similarity between Chinese and Spanish, as well as between different times of imitation within a language (i.e., t1 and t2, t2 and t3, t1 and t3) using the CoSMoMVPA toolbox (http://www.cosmomvpa.org/). Specifically, brain responses for each trial were estimated using unsmoothed preprocessed data. The Least-Squares Separate (LSS) method (Mumford et al., 2012) was used, with six head movement parameters and all other trials as covariates. A searchlight approach was adopted, where a sphere containing 125 voxels was centered at each voxel and moved across the entire brain. For the cross-language similarity analysis, pattern similarity between Chinese and Spanish was calculated using split-half correlation. Specifically, within each searchlight, a Pearson’s correlation coefficient was calculated between each Chinese trial and each Spanish trial on the beta values of the 125 voxels, and in total there were 84 × 84 = 7056 such correlation coefficients, because we had 84 trials (28 × 3 = 84) in each language. Then, we averaged these 7056 correlation coefficients to represent similarity between Chinese and Spanish at this voxel.

For the similarity between different times of imitation within a language, at each searchlight, a Pearson’s correlation coefficient was calculated between each trial and each other trial in the same imitation order within a language (28 trials in total) on the beta values of the 125 voxels; therefore, we had a 28 × 28 DSM. Spearman’s correlations were calculated between the DSMs of the first imitation and the second imitation, between the second and the third imitation, and between the first and the third imitation. Following the analysis, the results were z-transformed and subjected to further group analysis. The threshold was set at uncorrected *p* < .001 at the voxel level and FWE-corrected *p* < .05 at the cluster level when reporting group analysis.

#### 2.5.4 Machine learning

In order to confirm that the three groups have different representation patterns of foreign speech, we adopted a machine-learning approach. After calculating the representational similarity between different times of imitation, three machine-learning models were set up to classify children versus control adults, children versus adults with singing training, and control adults versus adults with singing training separately using similarity between t1 and t2, t2 and t3, t1 and t3 in each language for both perception and production. In total, there were 12 similarity parameters (i.e., three similarities in two languages for both perception and production). The analysis was performed using PRoNTo v2.1 (Pattern recognition for neuroimaging toolbox) (http://www.mlnl.cs.ucl.ac.uk/pronto/prtsoftware.html). Specifically, using training data, the 12 similarity parameters were first averaged and then mean-centered. Subsequently, a binary linear Support Vector Machine (SVM) was employed to train the model in a whole-brain gray matter mask, with cross-validation performed using the leave-one-subject-out approach. To measure whether the classification is successful, 1000 permutations were conducted. For a successful classification, the contributing weight map was computed. The weight map was then thresholded at 30% of the maximum weight value with 100 extended voxels.

#### 2.5.5 Statistical analysis on brain activation

A general linear model (GLM) was constructed in SPM12 (Statistical Parametric Mapping, http://www.fil.ion.ucl.ac.uk/spm) after the data were smoothed with an isotropic Gaussian kernel of 4 mm full width half maximum (FWHM). For each participant, the preprocessed functional images from all sessions were entered into a GLM to estimate the whole-brain neural activities for the perception and production stage. In the group-level statistical analysis, we conducted flexible factorial ANOVAs of group (control adults, adults with singing training, children) by imitation orders (t1, t2, t3) separately for perception and production for each language in SPM (Gläscher & Gitelman, 2008). The main effects of order and group as well as the interaction between order and group were calculated. The threshold was set at uncorrected *p* < .001 at the voxel level and FWE-corrected *p* < .05 at the cluster level.

## 3 Results

### 3.1 Behavioral tests

There was a significant main effect of group for both phonological awareness tests (for the rhyming judgment test: *F*(2, 69) = 15.86, *p* < .001, partial *η²* = .261; for the initial sound deletion test: *F*(2, 69) = 18.239, *p* < .001, partial *η²* = .261). The Bonferroni-corrected post-hoc analysis showed that children had significantly lower scores on the pseudoword rhyming judgment test than control adults (*t*(69) = 5.603, *p* < .001, partial *η²* = .313) and adults with singing training (*t*(69) = 3.575, *p* = .017, partial *η²* = .156). Children also had lower scores on the initial sound deletion test than control adults (*t*(69) = 5.97, *p* < .001, partial *η²* = .341) and adults with singing training (*t*(69) = 4.14, *p* < .001, partial *η²* = .199). No differences were found between the two adult groups on the pseudoword rhyming judgment test (*t*(69) = 1.637, *p* = .238, partial *η²* = .037), or the initial sound deletion test (*t*(69) = 1.371, *p* = .389, partial *η²* = .026). For the working memory test of digit span, a significant group effect was found in the test of forward order (*F*(2, 69) = 5.908, *p* = .004, partial *η²* = .105), but not in the test of reversed order (*F*(2, 69) = 1.761, *p* = .179, partial *η²* = .078). A Bonferroni-corrected post-hoc test revealed greater digit span in adults with singing training than in children (*t*(69) = 3.345, *p* = .025, partial *η²* = .14) and in control adults than in children (*t*(69) = 2.514, *p* = .043, partial *η²* = .084). No significant difference between the two adult groups (*t*(69) = 1.761, *p* = .179, partial *η²* = .043) was found. For the AMMA test, the adults with singing training significantly outperformed the control adults (*t*(50) = 8.568, *p* = .005, partial *η²* = .595).

### 3.2 Vots

**Fig. 1.**
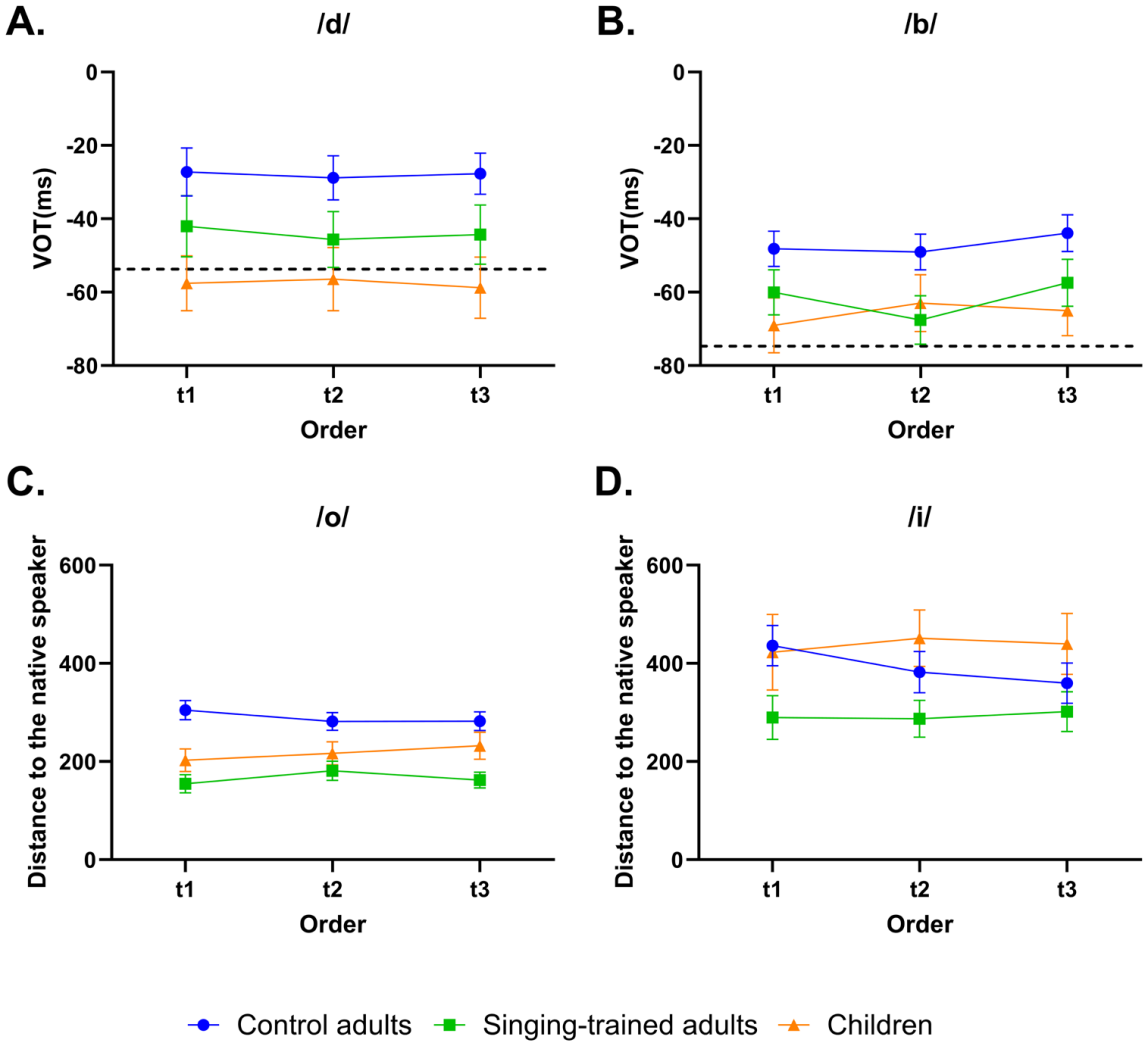
Control adults showed a poorer performance on VOT and vowel’s frequency formant than singing-trained adults and children. A and B are the VOT results for /d/ and /b/ in each group; C and D are the distance to the native speaker in the frequency formant space for /o/ and /i/. The dotted line in A and B is the VOT for /d/ and /b/ in a native Spanish speaker.

We ran an ANOVA of group by order for each consonant separately (i.e., /d/ and /b/). We found a significant main effect of group for /d/ (*F*(2, 65) = 5.299, *p* = .007, partial *η²* = .14) and /b/ (*F*(2, 65) = 3.445, *p* = .038, partial *η²* = .096). Bonferroni-corrected post-hoc analysis revealed that children’s VOT was more negative than control adults (*t*(45) = 3.934, *p* = .006, partial *η²* = .256) for /d/, and marginally more negative than control adults (*t*(45) = 2.847, *p* = .06, partial *η²* = .153) for /b/ (Fig. 1). Adults with singing training did not differ significantly from the other two groups in the VOTs of either /d/ (for comparison with children: *t*(33) = -1.857, *p* = .574, partial *η*² = .095; for comparison with control adults: *t*(48) = 1.724, *p* = .248, partial *η*² = .058) or /b/ (for comparison with children: *t*(33) = -.907, *p* = .645, partial *η²* = .024; for comparison with control adults: *t*(48) = 1.955, *p* = .184, partial *η²* = .074). The other main effects or interaction effects were not significant.

### 3.3 Formant of the vowels

To evaluate the imitation performance, we calculated the distance between the participant’s vowel in the frequency space (F1, F2) and the native speaker’s vowel (F1_stimulus_, F2_stimulus_). Then, a repeated-measure ANOVA of group by order was conducted for each vowel on the distance. For the vowel /o/ and /i/, the ANOVA revealed a significant main effect of group (*F*(2, 63) = 11.559, *p* < .001, partial *η²* = .187 for /o/, and *F*(2, 63) = 5.965, *p* = .004, partial *η²* = .101 for /i/). Bonferroni-corrected post-hoc analysis revealed that for /o/, control adults had a larger distance than adults with singing training (*t*(48) = 4.679, *p* < .001, partial *η²* = .314) and children (*t*(43) = 2.793, *p* = .013, partial *η²* = .153). For /i/, children and control adults had a larger distance than adults with singing training (for children: *t*(31) = 1.619, *p* = .007, partial *η²* = .078; for control adults: *t*(48) = 1.694, *p* = .019, partial *η²* = .056) (Fig. 1). However, no significant group difference was detected for the other vowels (/a/: *F*(2, 63) = 1.419, *p* = .250, partial *η²* = .064; /e/: *F*(2, 63) = .527, *p* = .593, partial *η²* = .007; /u/: *F*(2, 63) = .902, *p* = .411, partial *η²* = .09). No main effects of order or interactions between group and order were significant for any vowel.

### 3.4 Representational similarity analysis

#### 3.4.1 Similarity between Chinese and Spanish

**Fig. 2.**
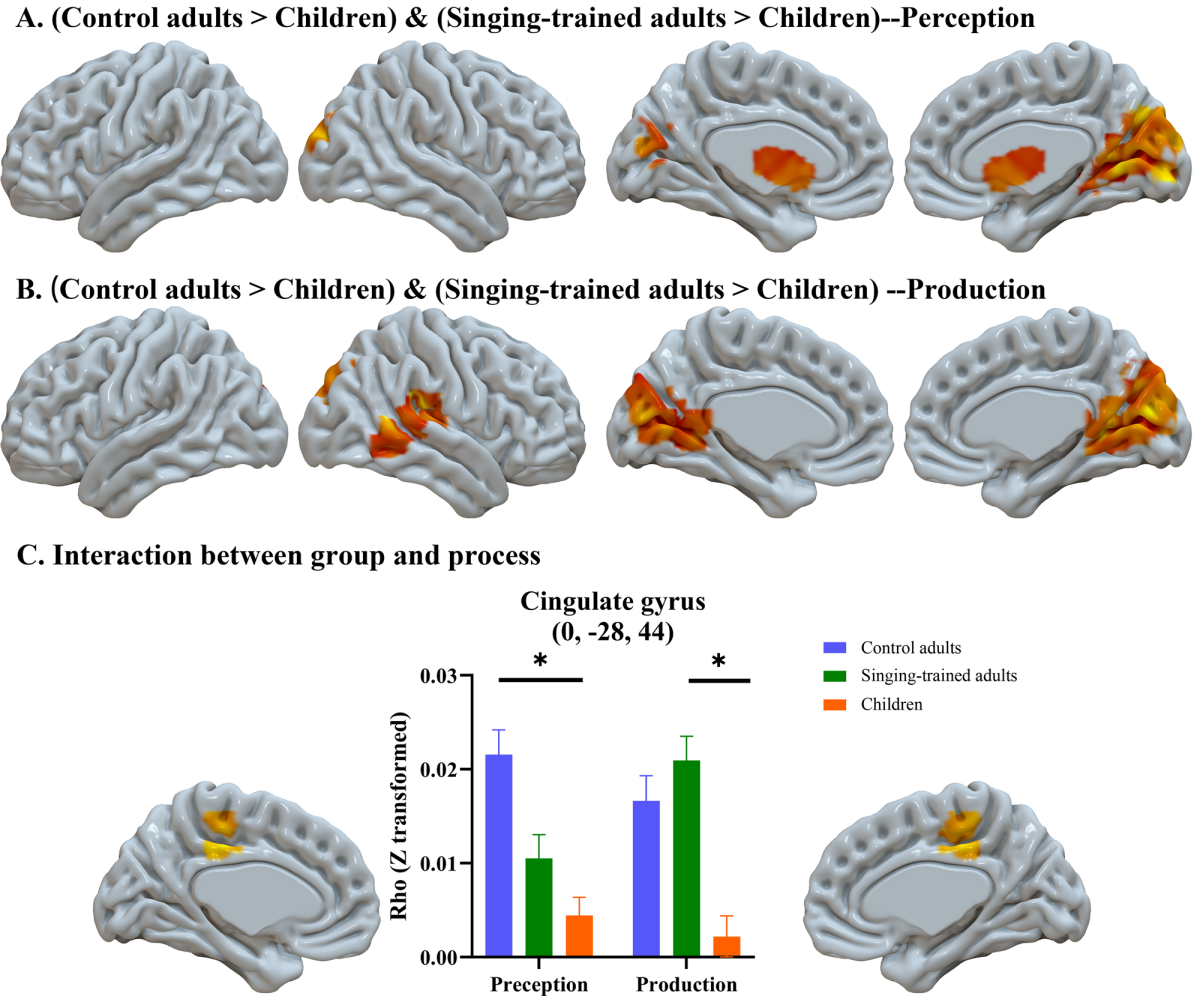
Results of the similarity between Chinese and Spanish. Adults showed greater similarity between the two languages than children in both the perception and production processes, while children did not show greater similarity than adults (A, B). (C) is the interaction between group and process for the similarity between Chinese and Spanish. Adults with singing training showed greater similarity in the cingulate gyrus in production than perception, while control adults showed greater similarity in perception than production.

We conducted an ANOVA of group by process (perception, production) to examine group differences in the similarity between the two languages during perception and production. We found main effects of group and processes, as well as interactions between group and process. For the group differences, we found that both adult groups showed greater similarity than children, but children did not show greater similarity than adults; therefore, we conducted a conjunction analysis between control adults>children and adults with singing training>children. The conjunction analysis showed that both control adults and adults with singing training showed greater representational similarity between Chinese and Spanish than children in the bilateral caudate/thalamus and superior occipital gyrus, cuneus during perception, and in the bilateral precuneus, cuneus, posterior cingulate and right STG during production (Table 2, Fig. 2A, B). For the main effect of process, we found greater similarity in the bilateral thalamus and caudate in perception than in production (Table 2).

**Table 2.** Results of the cross-linguistic similarity.

			MNI coordinate			
Anatomical label	H	Cluster size (Voxels)	x	y	z	Z
(Control adults>children) & (adults with singing training>children) (Perception)						
Superior occipital gyrus/cuneus, BA 7/17/18/19	B	703	18	-84	18	3.71
Caudate/ Thalamus	B	145	4	14	0	3.48
(Control adults>children) & (adults with singing training>children) (Production)						
Precuneus/ posterior cingulate gyrus, BA 18/30	L	125	-20	-52	2	3.42
Calcarine sulcus/cuneus/posterior cingulate gyrus, BA7/18/19/23	B	1265	20	-50	6	4.07
Rolandic/ superior temporal gyrus/middle temporal gyrus, BA 13/22/40/41	R	299	54	-28	20	3.44
Perception>production (main effect)						
Thalamus, caudate	L	131	-14	-18	14	4.47
Brain stem	R	138	0	-22	-8	4.42
Thalamus, caudate head	R	57	0	6	2	4.19
Caudate body	L	59	-8	14	10	3.99
Interaction of group by process (perception, production)						
Cingulate gyrus/supplementary motor area, BA 31	B	138	0	-28	44	4.92

There was an interaction between group and process in the cingulate gyrus/SMA, driven by greater cross-linguistic similarity in control adults than in children in perception, and greater similarity in adults with singing training than in children in production (Fig. 2C).

#### 3.4.2 Similarity between the 1^st^, 2^nd^, and 3^rd^ imitation

**Fig. 3.**
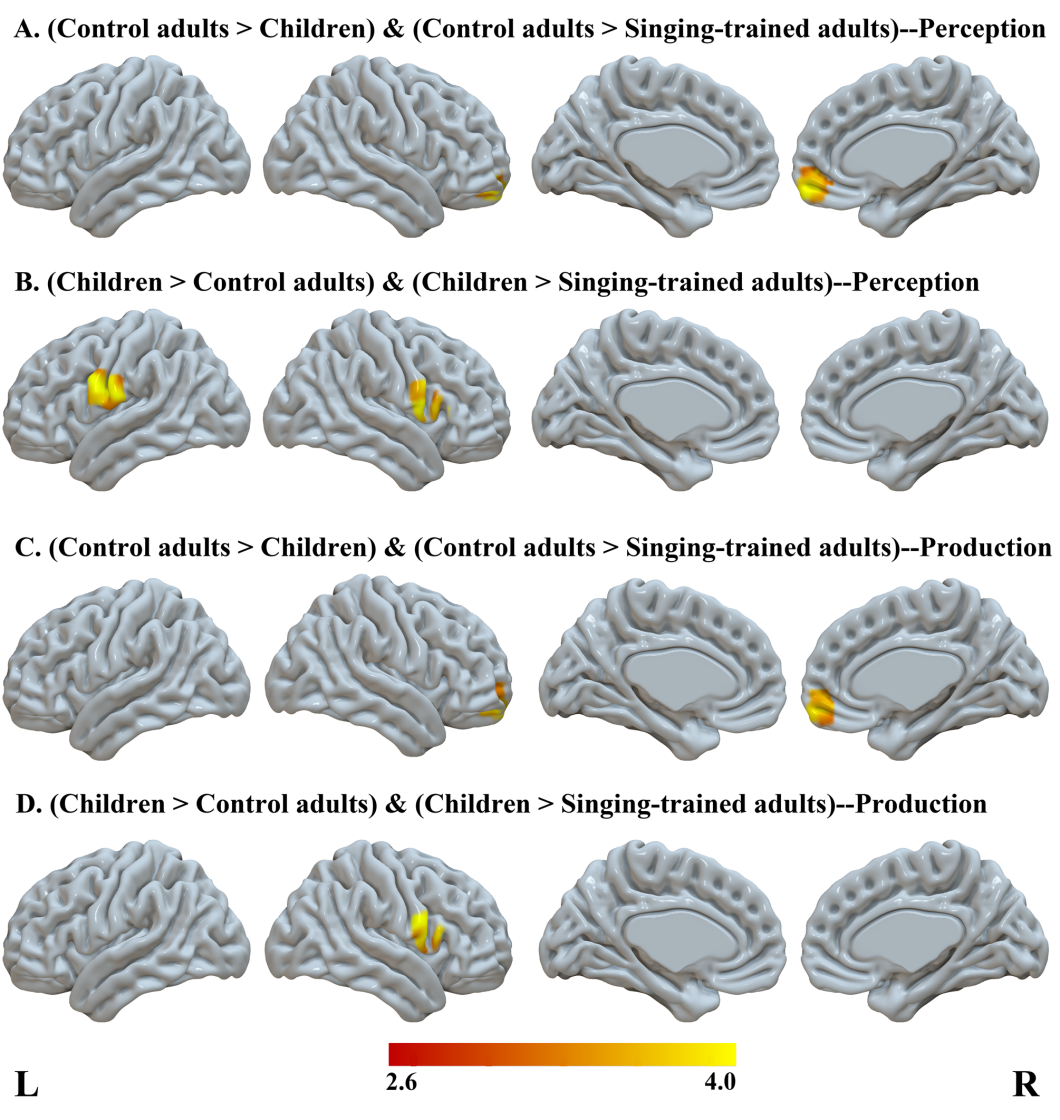
Results of the similarity between different times of imitation. We conducted conjunction analysis on the main effect of group across both Chinese and Spanish to find specific effects for control adults in perception (A) and production (C), and for children (B, D); however, we did not find specific effects for adults with singing training.

We also calculated representational similarity between different times of imitation within a language separately for perception and production. Then, we conducted a group (3) by language (2) ANOVA separately for perception and production. We found main effects of group. Then, we conducted a conjunction analysis to identify group-specific similarity patterns. Specifically, a conjunction between control adults>children and control adults>adults with singing training would reveal regions specific for control adults. A conjunction between children>control adults and children>adults with singing training would reveal regions specific for children. A conjunction between adults with singing training>children and adults with singing training>control adults would reveal regions specific for adults with singing training. We found greater similarity in control adults than in children and adults with singing training in the bilateral medial orbital frontal cortex across both perception and production; and greater similarity in children than in control adults and adults with singing training in the bilateral inferior premotor/postcentral gyrus in both perception and production (Table 3, Fig. 3). In the conjunction analysis, we did not find greater similarity in adults with singing training than the other two groups.

**Table 3.** Results from the ANOVA of group by language for the similarity between different times of imitation.

			MNI coordinate			
Anatomical Label	H	Cluster size (voxels)	*x*	*y*	*z*	Z
(Control adults>children) & (control adults>adults with singing training) (Perception)						
Medial frontal gyrus superior frontal gyrus/rectus, BA 11	R	83	8	56	-12	4.97
(Children>control adults) & (children>adults with singing training) (perception)						
Postcentral gyrus/precentral gyrus, BA 6	L	46	-64	-4	26	Inf
Inferior frontal gyrus/precentral gyrus/rolandic, BA 44	R	56	60	12	6	5.31
(Control adults>children) & (control adults>adults with singing training) (Production)						
Medial frontal gyrus/superior frontal gyrus, BA 10	R	90	12	56	-10	4.72
(Children>control adults) & (children>adults with singing training) (production)						
Rolandic operculum/precentral gyrus/inferior frontal gyrus, BA 44	R	52	60	10	13	4.90
Spanish>Chinese (perception)						
Thalamus	L	45	-8	-24	10	4
Interaction between group and language (Perception)						
Superior temporal gyrus/Heschl’s gyrus, BA 22	L	46	-58	-12	6	4.20
Supramarginal gyrus/inferior parietal lobule, BA 40	L	62	-56	-42	36	4.72
Interaction between group and language (Production)						
Insula/postcentral gyrus/supramarginal gyrus, BA 40	L	42	-50	-22	14	4.13
Superior temporal gyrus/rolandic operculum/transverse temporal gyrus, BA 41, 42	L	128	-46	-26	14	4.27

For the main effect of language, we found greater similarity in Spanish than in Chinese in bilateral thalamus during perception (Table 3).

**Fig. 4.**
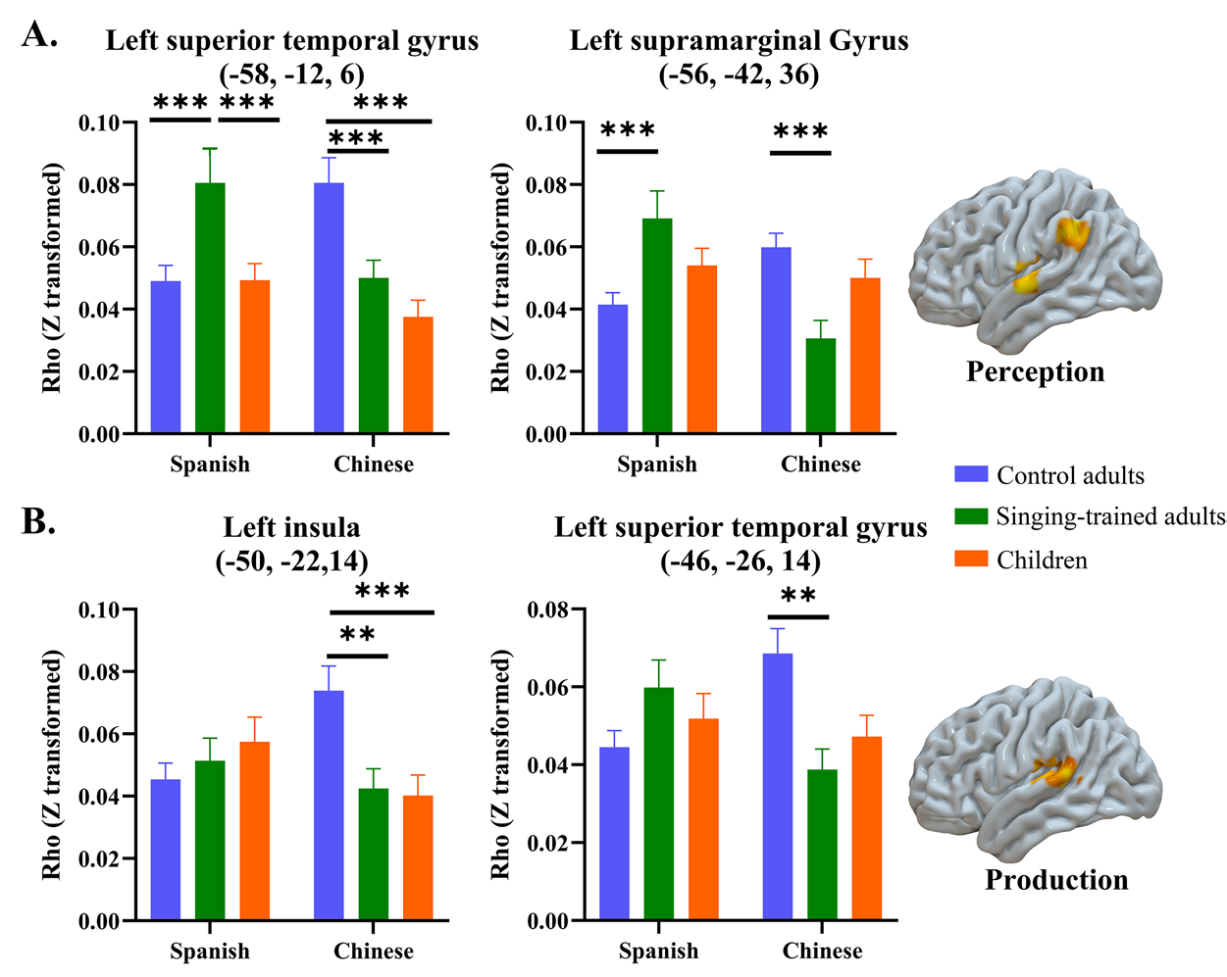
The interaction between group and language for the perception (A) and production process (B) in the similarity between different times of repetition. **p < 0.01; ***p < 0.001.

We found interaction effects between group and language for both perception and production (Fig. 4). For perception, we found interactions at the left STG and supramarginal gyrus. At the left STG, adults with singing training had greater similarity than the other two groups in Spanish, while control adults had greater similarity than the other two groups in Chinese. At the left supramarginal gyrus, adults with singing training had greater similarity than control adults in Spanish while control adults had greater similarity than adults with singing training in Chinese. Children did not show difference from the other two groups in either language at the left supramarginal gyrus.

For production, control adults showed greater similarity than the other two groups in the left insula in Chinese, but no group differences were found in Spanish. In the left STG, control adults showed greater similarity than adults with singing training in Chinese, but no group differences were found in Spanish.

#### 3.4.3 Machine learning results

**Fig. 5.**
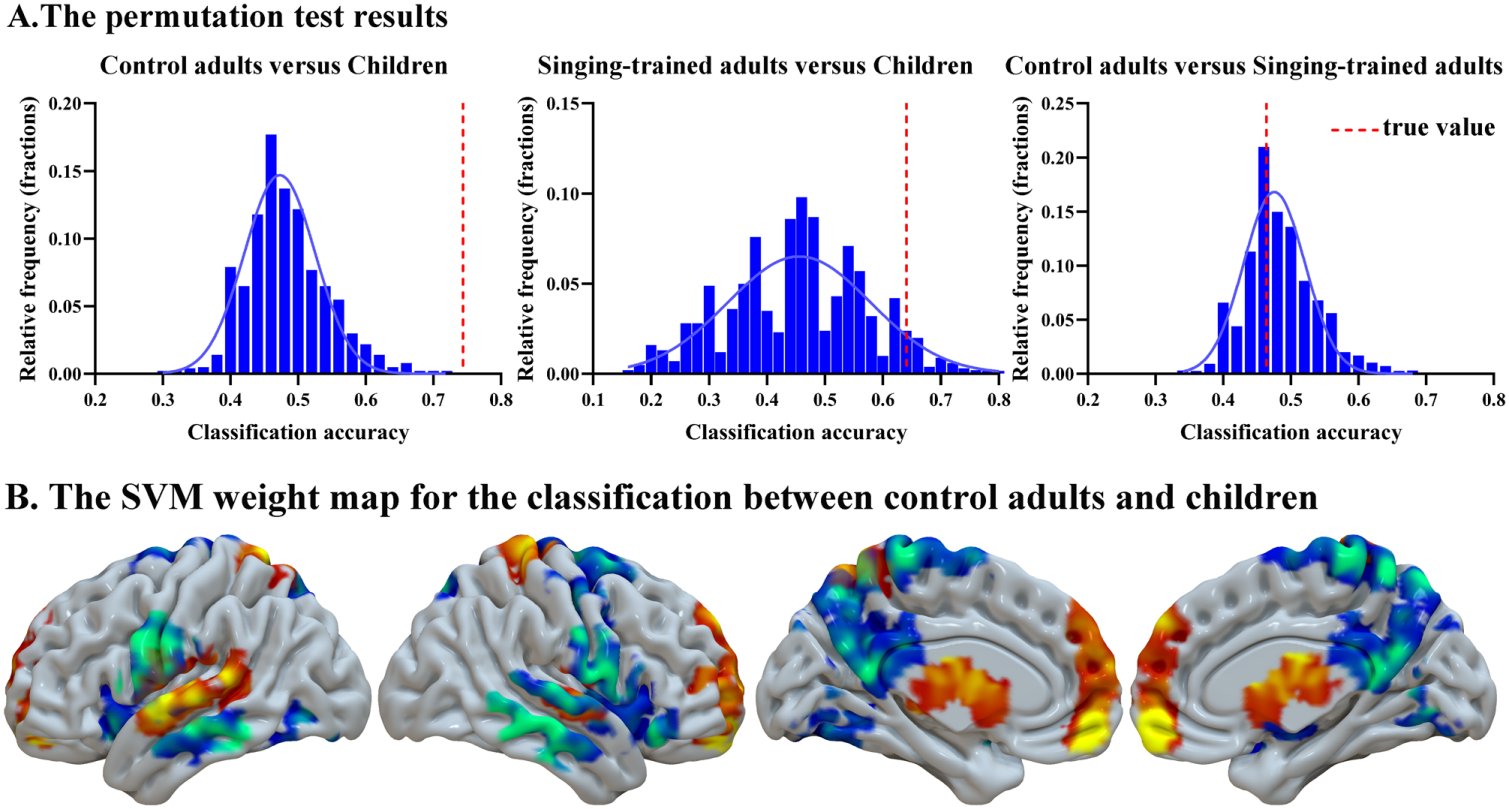
Machine learning results for the classifications. (A) Permutation results for the three classifications. (B) The SVM weight map for the classification between control adults and children. The warm color shows regions with a significant contribution to the contrast of control adults minus children; the cool color shows regions with a significant contribution to the contrast of children minus control adults.

Machine learning yielded significant results when classifying control adults and children (ACC = 74.38%, *p* = .001) (Fig. 5). The bilateral medial frontal gyrus, bilateral superior temporal gyrus, bilateral caudate/thalamus, and bilateral postcentral gyrus/precentral gyrus contributed to the contrast of control adults minus children; in contrast, the bilateral superior frontal gyrus, bilateral precentral/ postcentral gyrus/precuneus, bilateral posterior cingulate gyrus, bilateral insula/Rolandic region, bilateral middle temporal gyrus, and bilateral cerebellum contributed to the contrast of children minus control adults. Classification between control adults and adults with singing training (ACC = 46.38%, *p* = .675) or between children and adults with singing training (ACC = 64.08%, *p* = .059) did not yield any significant results.

#### 3.4.4 Brain-behavioral correlation

At the whole-brain level, we correlated brain similarity (i.e., both cross-linguistic similarity and similarity between different times of imitation within a language) with VOT and frequency separately in each group with a mask of brain regions that showed a significant main effect or interaction in the cross-linguistic similarity and similarity between different times of imitation analyses. We also regressed out working memory as measured by digit span in the correlation analysis, because digit span showed a negative correlation with the distance to the native speaker in VOT of /b/ in Spanish imitation (r = -.4, *p* = .005), suggesting higher digit span with higher performance. In the brain behavioral correlation analysis, we used distance to the native speaker in the VOT and frequency measures. We calculated a PCA score for VOT and frequency separately across multiple consonants and vowels to simplify the analysis.

**Fig. 6.**
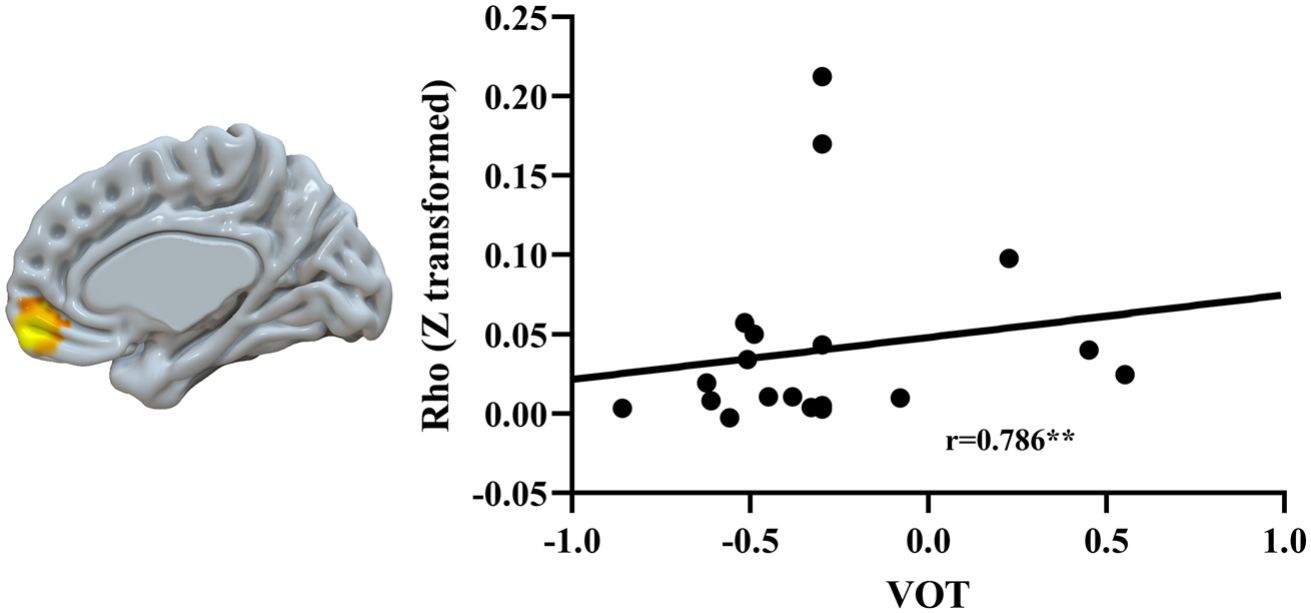
Brain behavioral correlation. At the right medial orbital frontal area, greater similarity between different times of Spanish imitation in children during perception was correlated with greater PCA factor score on VOT, indicating lower performance. This same region also showed greater similarity between different times of imitation in control adults than the other two groups during both perception and production.

We found that children showed a positive correlation between similarity of the 1^st^ and 2^nd^ imitation of the Spanish word during perception and VOT distance to the native speaker in the right medial orbital frontal cortex. At exactly the same region, we found greater similarity in control adults than the other two groups between different times of imitation (Fig. 6). It suggests that greater similarity in this region is correlated with lower performance in Spanish imitation in children. We did not find correlation in the other groups and similarities.

### 3.5 Brain activation analysis

#### 3.5.1 Interaction effects

**Fig. 7.**
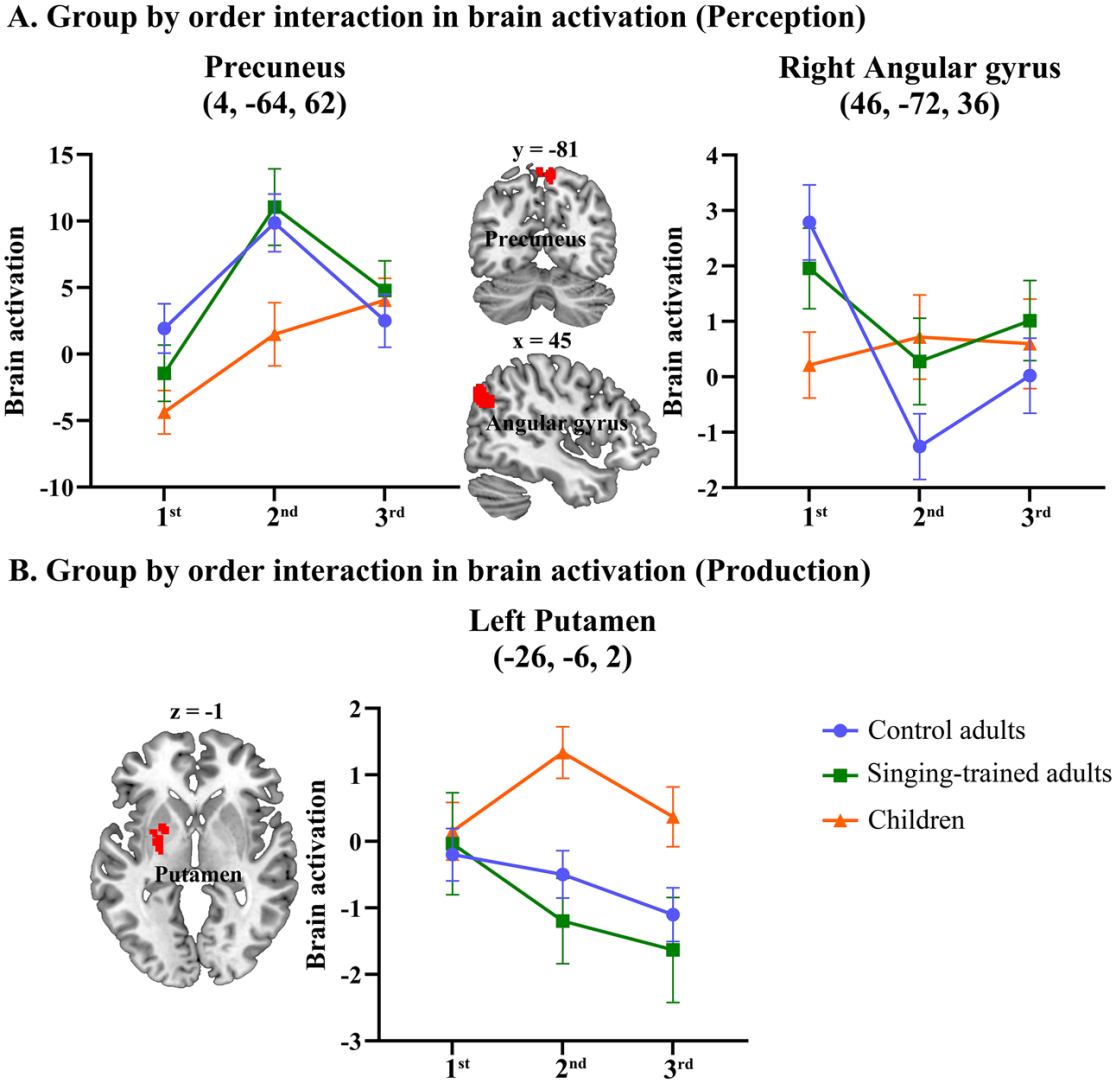
Interaction effects between group and order in brain activation analysis for Spanish imitation. Children showed different patterns than the other two adult groups at the right precuneus and right angular gyrus during Spanish perception and in the left putamen during Spanish production.

A flexible factorial ANOVA of group by order was conducted in Spanish for perception and production separately to understand the brain mechanisms of foreign speech learning in different groups. We only report interaction effects between group and order. In Spanish perception, two regions showed significant interactions between group and imitation order: bilateral precuneus and right angular gyrus. We further extracted BOLD signal percentage change at each imitation for each participant and drew line graphs to explain what drove the interaction. Children showed less reduction of brain activation from t1 to t2 than the two adult groups in the right angular gyrus. In bilateral precuneus, children showed less increase at t2 than the two adult groups. In Spanish production, we found an interaction in the left putamen, due to less reduction at t2 in children than the adult groups (Table 4, Fig. 7).

**Table 4.** Brain regions with a significant interaction between group and order in Spanish in brain activation analysis.

			MNI coordinate			
Anatomical label	H	Cluster size (voxels)	*x*	*y*	*z*	Z
Interaction between group and order (perception)						
Precuneus, BA 7	B	112	4	-64	62	3.73
Angular/precuneus/middle occipital gyrus, BA 19/39	R	133	46	-72	36	4.61
Interaction between group and order (production)						
Putamen/pallidum, BA 34	L	129	-26	-6	2	4.17

MNI: Montreal Neurological Institute space.

## 4 Discussion

In this study, we aimed to examine if children and adults with singing training have more advantages than control adults in foreign speech imitation, and the underlying neural mechanisms of this phenomenon. By analyzing consonants’ VOT and vowels’ frequency formants, we found that children and adults with singing training have better performance than control adults in foreign speech imitation, but their advantages were not consistent for all speech sounds. Furthermore, representational similarity analysis on fMRI data suggests that adults demonstrate greater similarity between the foreign speech and native speech representation in the brain during both perception and production than children, supporting the assimilation hypothesis of the critical period effect. Furthermore, similarity patterns between different times of imitation can be used to classify children and control adults, but it was not successful in the classification of adults with singing training from children or control adults. The three groups revealed similarities in different brain regions. Specifically, control adults showed greater similarity in the medial orbital frontal cortex than the other two groups; children showed greater similarity in the bilateral inferior premotor/postcentral gyrus than the other two groups; adults with singing training had greater similarity in the left STG than the other two groups during Spanish perception. To our knowledge, these findings provide the first neuroimaging evidence and important insights in understanding why children and adults with singing training have advantages than control adults in foreign speech learning.

### 4.1 The similarity between Chinese and Spanish

We found that both adult groups had greater similarity between Chinese and Spanish than children in the bilateral precuneus and cuneus during both perception and production. Children did not show greater similarity in any part of the brain than adults. This is consistent with the hypothesis that adults tend to assimilate foreign speech sounds to native speech sounds when learning a new language (Schwartz & Sprouse, 1996). In other words, they use the existing L1 speech representation system to perceive and produce foreign speech sounds. On the contrary, children have the ability to develop new categories for foreign speech sounds and coordinate articulators to learn new motor control for making foreign speech sounds. Taken together, we show direct evidence for greater neural similarities in the perception and production of native and foreign speech sounds in adults than in children, which may explain why adults tend to have a stronger accent when learning a foreign speech than children.

Moreover, a main effect of process was found at the bilateral thalamus and caudate across all groups, with greater similarity between the two languages in perception than production, suggesting that these two regions are involved in a similar way for native and foreign speech perception; however, for speech production, these two regions differentiate native from foreign speech. This might be because production needs more accurate and differentiated motor control for native and foreign speech than perception. Furthermore, at the cingulate gyrus/SMA, control adults showed greater similarity between the two languages than children in perception, while adults with singing training showed greater similarity than children in production. This suggests that music training helps to sharpen foreign speech perception in adults in these regions so that adults with singing training showed similar differentiation between native and foreign speech as children in these regions during perception. However, during production, adults with singing training did not show as much differentiation between the two languages as children in these regions, suggesting that production may be more influenced by age of acquisition which cannot be compensated by music training.

### 4.2 The similarity between the 1^st^, 2^nd^, and 3^rd^ imitations

Across both perception and production, control adults have a greater similarity of brain activation patterns between different times of imitations than children and adults with singing training in the bilateral medial orbital frontal cortex. Children, on the other hand, showed greater similarity in the bilateral inferior premotor/postcentral gyrus than the other two groups. These findings suggest that there are specific similarity patterns in the two groups. The medial orbital frontal cortex is involved in adaptive, flexible behavior in the face of challenging and unexpected outcomes (Gourley et al., 2016; Schoenbaum et al., 2009). In terms of foreign speech learning, the sounds produced may be different from the expected outcome, which requires constant adaptive learning, leading to the activation of the medial orbital frontal cortex. Control adults showed greater similarity in this region than the other two groups, presumably because the speech sounds they produced are different from their expectations, making their reliance of this adaptive learning mechanism to a greater degree. Furthermore, in the brain behavioral correlation analysis, we found that children who showed greater similarity between different times of imitation in this region tended to have a lower performance in the Spanish imitation task, suggesting that this adaptive learning mechanism might be employed by low performers.

Children showed specific similarity in the bilateral inferior precentral/postcentral gyrus compared to the other two groups. The postcentral gyri are involved in somatosensory feedback during speech production according to the DIVA model (Tourville & Guenther, 2011). The inferior precentral gyrus is involved in lip and tongue movement control (Brown et al., 2008), and serialization (Flinker et al., 2015; Shuster & Lemieux, 2005) during speech output. These speech motor control regions and somatosensory feedback regions that support sensorimotor learning are more consistently involved in different times of imitation in children than in adults, which may explain why children perform better than adults in foreign speech imitation.

Adults with singing training, on the other hand, showed greater similarity between different times of Spanish perception in the left STG than the other two groups (Fig. 4). Previous studies have found that extensive musical experience significantly changes brain structure and function in the auditory cortex, including increased grey matter volume (Gaser & Schlaug, 2003) and increased brain function (Du & Zatorre, 2017). The auditory cortex is essential in speech perception and auditory feedback during speech production (Bamiou et al., 2003). Greater similarity in the STG in adults with singing training than the other two groups suggests that musical training increases the reliance on auditory perception during foreign speech learning in singers.

From a machine-learning perspective, the similarities between different times of imitation can be used as predictive classification of children from control adults, with an accuracy of 74%, suggesting different representational patterns in these two groups. However, machine learning is less successful in classifying adults with singing training from the other two groups. This suggests clear distinction between children and control adults in the neural mechanisms of speech learning. However, singing training somehow modulates the neural mechanisms in adults so that adults with singing training are not distinguishable from children in the brain, even though they are not distinguishable from control adults either.

In addition, we found a main effect of language during perception across all three groups in the similarity between different times of perception with greater similarity in Spanish than in Chinese in the thalamus. The thalamus is an important subcortical center for sensory relay (Torrico et al., 2023). This suggests that the thalamus shows greater stability for more challenging tasks, such as when less familiar speech sounds are perceived, than when native language is perceived.

### 4.3 Brain activation

In the brain activation analysis, we found less repetition suppression in children than in adults from the first time to the second time of imitation in the right angular gyrus during Spanish perception and in the left putamen during Spanish production. Less repetition suppression in children than in adults may reflect more learning and adjusting during foreign speech imitation in children than in adults. The greater reduction in the putamen in adults than in children is consistent with our expectation that the vocal learning pathway (striatum, thalamus and premotor) is inactive too early in adults, which may explain the foreign accent. This is also consistent with the model by Jarvis (2006) and Simmonds (2015).

At the precuneus, adults showed greater increase from t1 to t2 than children. The activation in the precuneus was negative at t1, which indicates that this region may serve as part of the default mode network (DMN) (Raichle, 2015). Therefore, increase in this region may suggest less effort at t2. Children showed less increase at t2 compared to adults, implicating that they are still implementing cognitive effort at t2. Taken together, the interaction effects in brain activation suggest more persistent neural adjustment and learning from t1 to t2 in children than in adults.

Reduced repetition suppression in the right angular gyrus and left putamen and less increase in the DMN in children may also indicate greater neural variability (Waschke et al., 2021), which may explain why children outperform adults in learning a second language. According to the neural sampling theory, neural variability encodes uncertainty of perceptual inferences (Haefner et al., 2016). Previous studies have found that BOLD variability decreases from childhood to young adulthood (Nomi et al., 2017), as well as from young adulthood to old adulthood (Garret et al., 2011, 2013). In addition to the developmental changes of neural variability, previous research has also found skill-related changes in neural variability. For example, a study found lower variability in traumatic brain injury patients than controls and increased brain signal variability is correlated with improved behavioral performance in all participants (Raja Beharelle et al., 2012). Another study found that older, slower, and more inconsistent performers showed lower BOLD signal variability across three different tasks (Garret, 2011). Taken together, greater neural variability in children than in adults may be associated with greater learning ability in many domains in children than in adults, including foreign speech imitation.

## 5 Conclusions

In this study, we showed clear evidence that children and adults with singing training have better performance in foreign speech imitation than control adults through VOT and frequency formant analysis. Through RSA and machine learning, we found possible neural mechanisms to explain the advantages of children and adults with singing training. First, children had lower representational similarity between native language and foreign language imitation than adults, suggesting that children are better at differentiating and accurately representing foreign speech sounds than adults. Second, similarity patterns between different times of imitation can be used to classify children from control adults, but not adults with singing training from children or control adults. Specifically, control adults showed greater similarity in the medial orbital frontal cortex, implicating adaptive learning; children showed greater similarity in bilateral inferior premotor/postcentral gyrus, suggesting sensorimotor learning; and adults with singing training showed greater similarity in the left STG, suggesting reliance on auditory feedback. Taken together, these findings pave the way for understanding why adults confront greater challenge in foreign speech learning than children, and how singing training may help.

## Supplementary Material

Supplementary Material

## Data Availability

All data and code will be available after acceptance of the paper based on request sent to the correspondence author with a possibility of needs for a formal data-sharing agreement, and approval from the requesting researcher’s local ethics committee.
