# Supplementary material for: Neural correlates of foreign speech imitation: The effects of age and music
Source: Imaging Neurosci (Camb). 2025 Jul 17;3:IMAG.a.75. doi: 10.1162/IMAG.a.75 (PMC12330863; doi:10.1162/IMAG.a.75)
Supplement: Supplementary Material [file IMAG.a.75_supp.pdf]

Supplementary materials

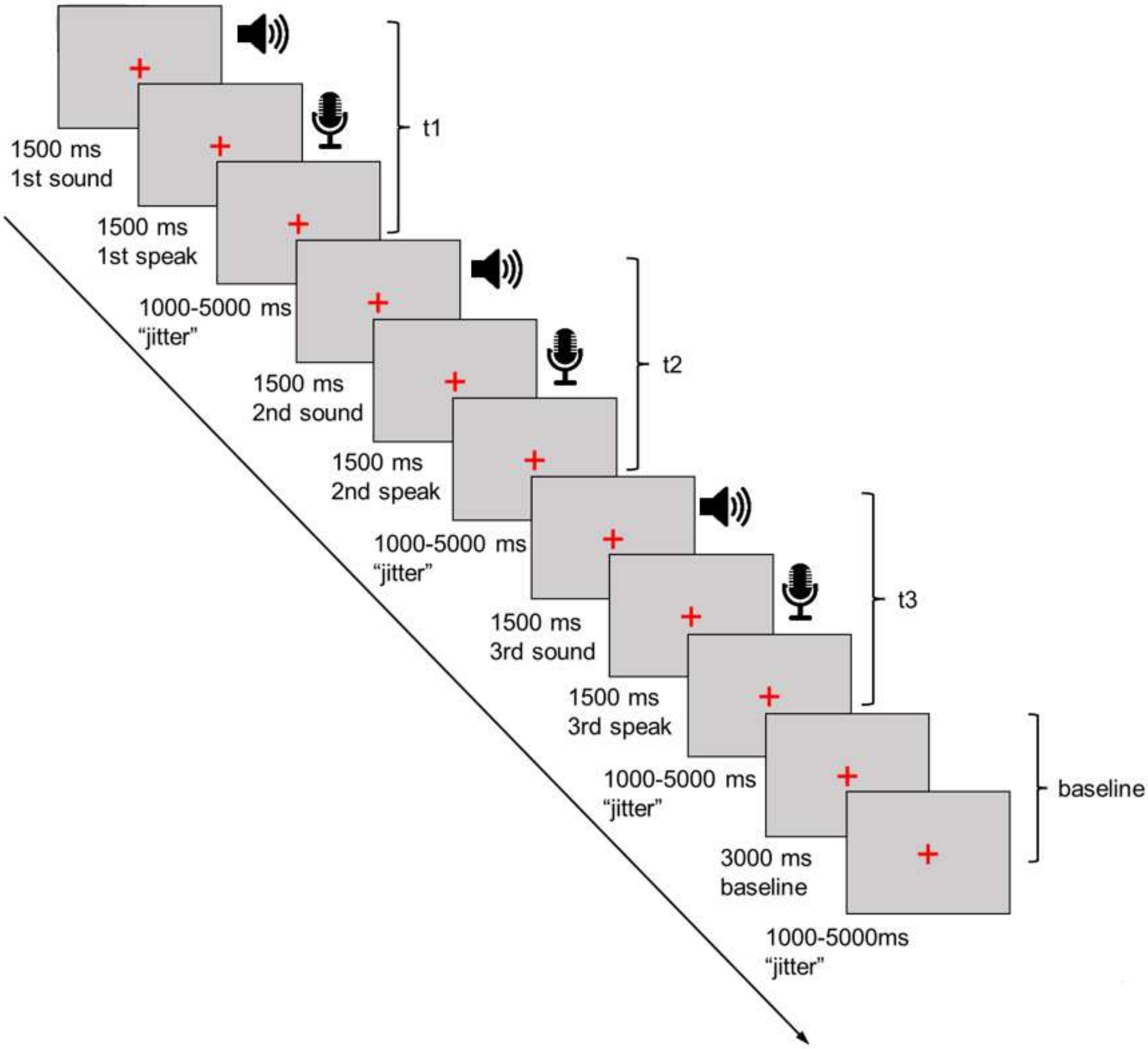

Figure S1. Procedures in the Speech Imitation Task. *Note.* t1 = the 1st imitation; t2 = the 2nd imitation; t3 = the 3rd imitation.

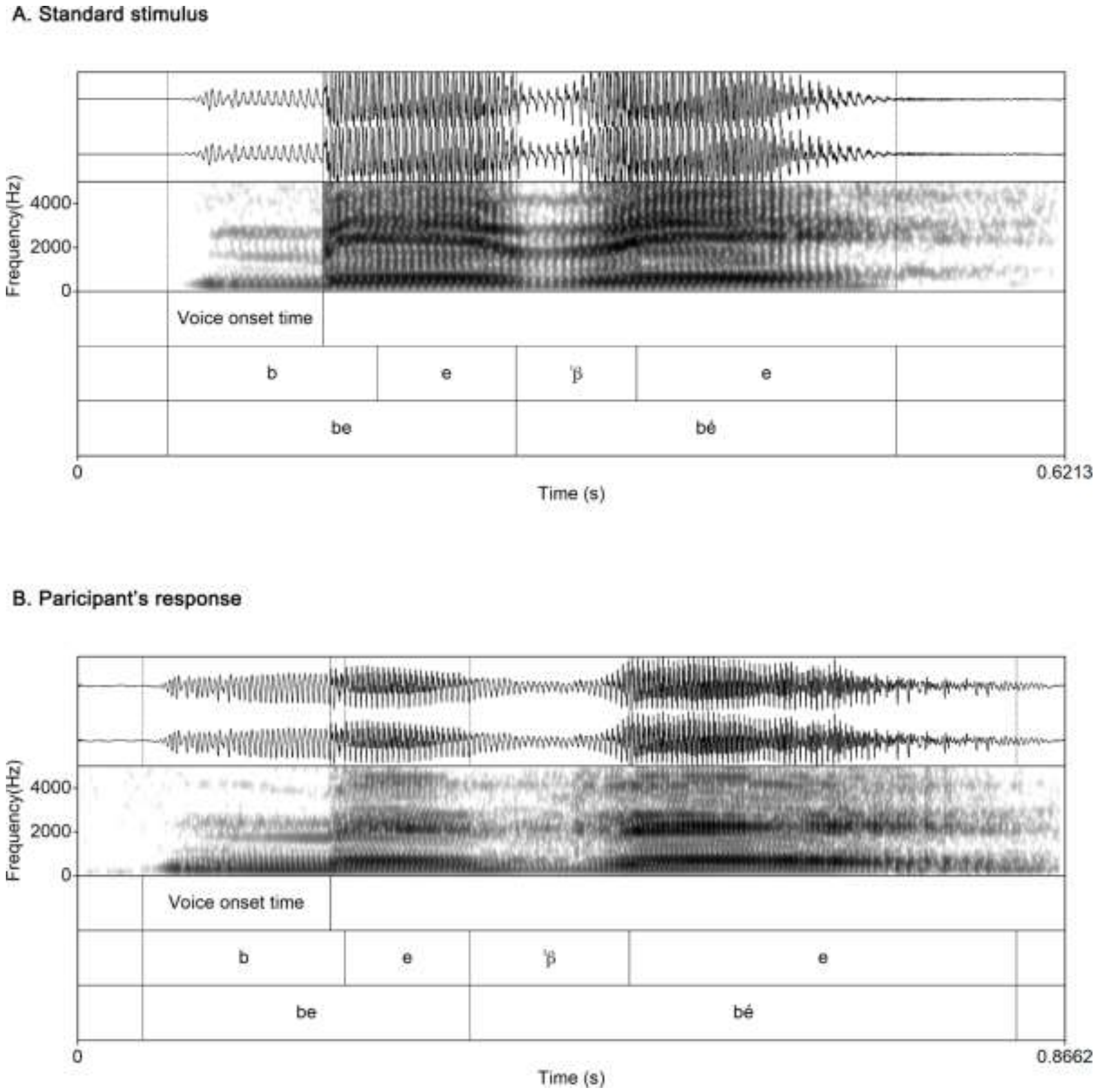

Figure S2. Example of Voice Onset Time (VOT) Measurement. (a) The waveform and spectrogram of the standard "bebé" from a native speaker; (b) The waveform and spectrogram of "bebé" from a first imitation of one participant.
